# Atypical Climacteric and Functional Ethylene Metabolism and Signaling During Fruit Ripening in Blueberry (*Vaccinium* sp.)

**DOI:** 10.3389/fpls.2022.932642

**Published:** 2022-06-23

**Authors:** Yi-Wen Wang, Tej P. Acharya, Anish Malladi, Hsuan-Ju Tsai, D. Scott NeSmith, John W. Doyle, Savithri U. Nambeesan

**Affiliations:** ^1^Department of Horticulture, University of Georgia, Athens, GA, United States; ^2^Center for Applied Genetic Technologies, University of Georgia, Athens, GA, United States; ^3^Taiwan Agricultural Research Institute Council of Agriculture, Taichung, Taiwan; ^4^Department of Horticulture, University of Georgia, Griffin, GA, United States

**Keywords:** respiration, ethylene, ripening, blueberry, fruit development

## Abstract

Climacteric fruits display an increase in respiration and ethylene production during the onset of ripening, while such changes are minimal in non-climacteric fruits. Ethylene is a primary regulator of ripening in climacteric fruits. The ripening behavior and role of ethylene in blueberry (*Vaccinium* sp.) ripening is controversial. This work aimed to clarify the fruit ripening behavior and the associated role of ethylene in blueberry. Southern highbush (*Vaccinium corymbosum* hybrids) and rabbiteye (*Vaccinium ashei*) blueberry displayed an increase in the rate of respiration and ethylene evolution, both reaching a maxima around the Pink and Ripe stages of fruit development, consistent with climacteric fruit ripening behavior. Increase in ethylene evolution was associated with increases in transcript abundance of its biosynthesis genes, *AMINOCYCLOPROPANE CARBOXYLATE* (*ACC*) *SYNTHASE1* (*ACS1*) and *ACC OXIDASE2* (*ACO2*), implicating them in developmental ethylene production during ripening. Blueberry fruit did not display autocatalytic system 2 ethylene during ripening as *ACS* transcript abundance and ACC concentration were not enhanced upon treatment with an ethylene-releasing compound (ethephon). However, *ACO* transcript abundance was enhanced in response to ethephon, suggesting that *ACO* was not rate-limiting. Transcript abundance of multiple genes associated with ethylene signal transduction was upregulated concomitant with developmental increase in ethylene evolution, and in response to exogenous ethylene. As these changes require ethylene signal transduction, fruit ripening in blueberry appears to involve functional ethylene signaling. Together, these data indicate that blueberry fruit display atypical climacteric ripening, characterized by a respiratory climacteric, developmentally regulated but non-autocatalytic increase in ethylene evolution, and functional ethylene signaling.

## Introduction

Fruit ripening involves coordinated progression of physiological and biochemical events that influence texture, flavor, color, and susceptibility to biotic and abiotic factors. Progression of ripening is associated with cell wall modifications, decrease in acidity, accumulation of sugars and pigments, and alterations in volatile profiles, parameters integral to final fruit quality (Coombe, 1976; Giovannoni, 2004). Hence, understanding the regulation of ripening is a key goal in fruit biology. Although all fleshy fruits display the ripening syndrome, they are often grouped into one of two categories, climacteric and non-climacteric. Climacteric fruits, such as tomato (*Solanum lycopersicum*), banana (*Musa ×* paradisiaca), and apple (*Malus* × *domestica* Borkh.) display an increase in the respiration rate (respiratory climacteric) and ethylene evolution concomitant with the initiation of ripening. In these fruits, ethylene is often the primary phytohormone regulating ripening-associated changes (Paul et al., 2012; Seymour et al., 2013). Ethylene is synthesized from methionine *via* the sequential activities of S-adenosyl methionine (SAM) synthetase (SAMS), 1-amino-cyclopropane carboxylate (ACC) synthase (ACS), and ACC oxidase (ACO). Ethylene synthesis has been proposed to operate under system 1 and system 2 mechanisms (McMurchie et al., 1972). System 1 ethylene is autoinhibitory and contributes to basal ethylene production in vegetative tissues and young fruit (Alexander and Grierson, 2002; Cherian et al., 2014). System 2 ethylene is autocatalytic and operates during flower senescence and climacteric fruit ripening (Alexander and Grierson, 2002; Cherian et al., 2014). Ethylene signaling is facilitated by multiple components leading from its perception by ethylene receptors to transcription factor mediated changes in gene expression. Multiple components of the ethylene-signaling pathway have been implicated directly in the regulation of ripening, particularly in tomato (Mcatee et al., 2015; Fenn and Giovannoni, 2021).

A respiratory climacteric and increase in ethylene evolution are either not discernable or not prominent in non-climacteric fruits, such as grape (*Vitis vinifera*), strawberry (*Fragaria × ananassa*), loquat (*Eriobotrya japonica* Lindl.), and citrus (*Citrus* sp.; Kays and Paull, 2004; Paul et al., 2012; Seymour et al., 2013). In these fruits, ethylene may not serve as the primary regulator but its production, sensing and signaling may yet be important in regulating specific aspects of ripening (Chervin et al., 2004; Trainotti et al., 2005; Alos et al., 2017). Other phytohormones such as abscisic acid (ABA) and auxin are proposed to play primary roles in regulating the progression of non-climacteric fruit ripening (Given et al., 1988; Moya-León et al., 2019; Fenn and Giovannoni, 2021).

The dogma of classification of fleshy fruits as climacteric or non-climacteric is blurred in some fruits. For example, melon (*Cucumis melo* L.) genotypes vary in their ripening behavior with some displaying climacteric and others, non-climacteric characteristics (Perin et al., 2002; Pereira et al., 2020). Similarly, Japanese plum (*Prunus salicina* Lindl.) genotypes display a wide range of climacteric/non-climacteric fruit ripening behavior (Minas et al., 2015). Kiwifruit (*Actinidia deliciosa*) is considered a climacteric fruit; however, initiation of ripening related changes occur before autocatalytic ethylene production (Mcatee et al., 2015; Asiche et al., 2018). Despite this, ethylene production from system 2 further accelerates softening and volatile production in kiwifruit, suggesting atypical ripening behavior (Richardson et al., 2011; Asiche et al., 2018).

Ripening physiology and its regulation remain poorly characterized in blueberry (*Vaccinium* sp.); a fruit crop with increased popularity due to consumer awareness of potential health benefits (Retamales and Hancock, 2012). Recent publications describe blueberry fruit ripening as climacteric (Colle et al., 2019; Yan et al., 2020), non-climacteric (Costa et al., 2018; Chung et al., 2019), or as controversial in its classification (Cappai et al., 2018; Watanabe et al., 2021). An increase in respiration and ethylene evolution during ripening was noted in northern highbush and rabbiteye blueberry cultivars suggesting a potential climacteric nature to the ripening process (Windus et al., 1976; El-Agamy et al., 1982; Shimura et al., 1986; Suzuki et al., 1997). Consistent with a climacteric fruit ripening response, expression of a gene coding for ACO, was greatly upregulated during later stages of ripening (Gupta et al., 2015). External application of the ethylene-releasing compound, ethephon, could accelerate progression of ripening (Eck, 1970; Dekazos, 1976; Ban et al., 2007; Wang et al., 2018; Colle et al., 2019). However, several other studies suggested non-climacteric fruit ripening in blueberry. In lowbush blueberry, the rate of respiration decreased gradually during fruit development until maturity without a notable change during ripening, suggesting a non-climacteric ripening process (Hall and Forsyth, 1967). Similarly, G-90, a selection of northern highbush blueberry, did not display a substantial climacteric rise in respiration or ethylene evolution during ripening (Frenkel, 1972). These studies highlight the lack of clear characterization of the ripening behavior in this fruit. Recently, an extensive literature review indicated that variability in ethylene evolution reported across previous studies could be attributed to the use of varying species and cultivars (Farneti et al., 2022). Further, evaluation of 12 highbush blueberry cultivars indicated genotype-dependent variation in the extent of ethylene evolution during fruit development, implying a potential role for ethylene in regulating blueberry ripening (Farneti et al., 2022). However, the potential role of ethylene metabolism and signaling in coordinating ripening requires further characterization in blueberry. Clarification of fruit ripening behavior and its potential regulation by phytohormones is essential to achieve advances in fruit quality improvement in blueberry. Hence, the main objectives of the current study were to determine ripening characteristics of the blueberry fruit and the role of ethylene in regulating its progression.

## Materials and Methods

### Fruit Collection and Determination of the Rate of Respiration

Fruit from six southern highbush and six rabbiteye cultivars was used for measurement of respiration rate over 2 years of study. In 2017, rate of respiration was determined in five southern highbush cultivars (“Emerald,” “Miss Alice Mae,” “Miss Lilly,” “Rebel,” and “Suziblue”) and six rabbiteye cultivars (“Alapaha,” “Brightwell,” “Krewer,” “Powderblue,” “Premier,” and “Titan”). In 2018, measurements were repeated for all cultivars evaluated in 2017 with two exceptions for the southern highbush cultivars: “Suziblue” could not be used and “Miss Jackie” was included. Fruit from above cultivars were collected from three commercial blueberry farms. In 2017, fruit from all cultivars were harvested at Cornelius Farms, Manor, GA, except for “Powderblue” and “Premier,” which were harvested at the Durham Horticulture Farm, Watkinsville, GA, United States. In 2018, “Emerald,” “Miss Alice Mae,” and “Rebel” fruit were collected from Cornelius Farms, Manor, GA, United States; “Miss Jackie,” “Miss Lilly,” “Alapaha,” “Brightwell,” “Krewer,” and “Titan” were collected from UGA Blueberry Research Farm, Alapaha, GA, United States; and “Powderblue” and “Premier” from Durham Horticulture Farm, Watkinsville, GA, United States.

Fruit were collected at various developmental, ripening, and postharvest stages. Early growth stages included S2, S3, and immature green—IMG (green fruit with diameter: 6–8, 8–10, and 10–12 mm respectively); while the ripening stages included green, pink, and ripe (predominantly green fruit with <10% pink; 100% pink; and 100% blue skin, respectively). Early growth stages collected were S3 for “Premier,” and S2 and S3 for “Powderblue.” These stages were collected during early fruit development, while the remaining stages were all collected on the same day when plants contained 30–50% ripe fruit. For fruit collected from Cornelius farms, Manor, GA, United States and the UGA Blueberry Research Farm, Alapaha, GA, United States, all fruit were brought to the laboratory and stored overnight in a walk-in cooler set at 4°C and ~90% relative humidity. The following day, fruit from all stages were sorted and kept at room temperature for several hours after which respiration and ethylene measurements were performed as described below. Additional ripe fruit were also re-sorted into clamshells and placed back in the walk-in cooler for measurements during postharvest stages between 8 and 26 days of storage. Fruit harvested from the Durham Horticulture Farm, Watkinsville, GA, United States were analyzed on the same day for respiration and ethylene measurements. Ripe fruit were sorted into clamshells and stored in the walk-in cooler as described earlier. Fruit respiration rates were determined by measuring CO_2_ production at various fruit developmental stages using a closed system. Approximately, 10 g of fruit were placed in an air-tight 495 ml glass jar fitted with a septum in the lid for 1 h at room temperature (23°C). Total fruit weight (g) was recorded before measurements. Headspace samples (60 ml) were extracted using a syringe and analyzed with a CO_2_ analyzer (Quantek, MA, United States, Model 902P). Rate of respiration was calculated as CO_2_ evolution (μl^.^g^-1.^h^−1^). Each stage of fruit development used four biological replicates.

### Determination of Ethylene Evolution

Five southern highbush and four rabbiteye cultivars were used for ethylene measurement in two years at fruit development and postharvest stages described above. Two southern highbush (“Miss Lilly” and “Suziblue”) and four rabbiteye cultivars (“Brightwell,” “Powderblue,” “Premier,” and “Titan”) were measured in 2017. Four southern highbush (“Emerald,” “Miss Jackie,” “Miss Lilly,” and “Rebel”) and four rabbiteye cultivars (“Brightwell,” “Powderblue,” “Premier,” and “Titan”) were measured in 2018.

Ethylene evolution from fruit was measured using a closed system. Approximately, 25 g of fruit were placed in an air-tight 135 ml glass jar with a lid fitted with a rubber septum, for 4 h. Headspace samples (1 ml) were analyzed using a GC-17A gas chromatograph (Shimadzu, Japan) equipped with a 2 m micropacked column (Hayesep N, Restek, PA, United States) and a flame ionization detector. The temperature of the injection port and the detector of GC were set at 200°C. The temperature program was: 60°C for 4 min; increased by 20°C˙min^−1^ to 150°C; and hold at 150°C for 1 min. Peak area from the resulting chromatograph and a standard curve generated using various concentrations of ethylene were used to determine ethylene evolution from the fruit sample and expressed as nl·g^−1^·h^−1^ (*n* = 4).

### Treatment With Ethylene

Fruit from the rabbiteye blueberry “Premier” at the Green and Pink stage and “Powderblue” at the Pink Stage were collected from the Durham Horticulture Farm, Watkinsville, GA, United States. Fruit were incubated in a glass jars with 0 ppm (control), 1 or 10 ppm ethylene and stored at 20°C for 18 h. After incubation, the jars were flushed with air and fruit were kept out for 30 min to equilibrate with room conditions. One set of fruit from each treatment was used to measure CO_2_ and ethylene as described above. The second set of fruit were placed in clamshells at 20°C for approximately 24 h after which they were allowed to equilibrate with room conditions for 30 min before ethylene and CO_2_ measurements were performed. Four and three replicates were used for “Premier” and “Powderblue,” respectively.

### Determination of ACC Content

Fruit from “Premier” and “Powderblue” rabbiteye blueberry collected in 2018 during fruit ripening (as described above) were used for these analyses. Fruit were transported to the laboratory, frozen in liquid nitrogen, and stored at −80°C. In addition, control and ethephon treated fruit of “Premier” and “Powderblue” in 2020 were collected from the same location. Spray applications were performed on individual blueberry plants when about 30–40% of fruit on the plant were ripe (blue). All pink and ripe fruit were removed and subsequently foliar application of control (surfactant only, 0.15% Latron B-1956) and ethephon (250 ppm along with surfactant), was performed after removing pink and ripe fruits on each plant. Fruit were collected at 0, 1, 2, and 3 days after the treatment from a single branch, and represented a mix of different developmental stages to allow for distinguishing between ethylene-induced and ripening-related changes. Fruit were immediately frozen in liquid nitrogen and stored at −80°C. Four biological replications were used for each stage and treatment.

Fruit were ground to fine powder using mortar and pestle in liquid nitrogen. Approximately, 2 g of finely ground sample was placed in a 15 ml Falcon tube and sample weight was recorded. The sample was resuspended in 4 ml of 5% sulfosalicylic acid buffer and thoroughly vortexed. Samples were gently mixed using a rocker for 30 min at 4°C. Next, samples were centrifuged at 4,500 rpm for 30 min and supernatant was transferred into a separate tube. Subsequently, 1.4 ml of the supernatant was transferred into a 20 ml vial (Restek Corporation, Bellefonte, PA) followed by the addition of 0.4 ml of mercuric chloride to the vial. The vial was immediately closed with a cap containing a septum. Next 0.2 ml of 2:1 sodium hypochlorite:sodium hydroxide mixture was introduced into to the vial by injecting with a needle and syringe. Samples were vortexed for 5 s and placed on ice for 4 min for the reaction to continue. During this time, ACC is non-enzymatically converted to ethylene. Samples were vortexed again for 5 s. Ethylene was measured by drawing out 1 ml of headspace gas using a syringe with needle and injecting into the GC as described above for ethylene measurements. To calculate the recovery of ACC (reaction efficiency), every sample was also spiked with 20 μl of 50 μM ACC and ethylene released was measured. On each day, three blank samples were also prepared, by using 20 μl of 50 μM ACC and measuring ethylene evolution after addition of mercuric chloride and 2:1 sodium hypochlorite: sodium hydroxide mixture. The reaction in the blank solution was assumed to have 100% reaction efficiency for conversion of ACC to ethylene. The concentration of ACC was calculated according to the protocol described by (Bulens et al., 2011).

### Quantitative RT-PCR

The samples used for quantitative RT-PCR were the same as those used for determination of ACC concentration. Transcript abundance of *ACS*, *ACO* and ethylene signaling genes were measured by quantitative PCR. Total RNA was extracted using a modified cetyltrimethylammonium bromide (CTAB)-based protocol (Vashisth et al., 2011). The synthesis of complementary DNA (cDNA) was conducted using 1 μg of total RNA. After removing potential DNA by DNase treatment, cDNA was synthesized by reverse transcription. Quantitative reverse transcription PCR (qRT-PCR) analysis was performed using PowerUp SYBR Green Master Mix (ThermoFisher, United States) reagent and with Stratagene Mx3005P quantitative real-time PCR instrument (Agilent Technologies, United States). Five *ACS* genes were identified from the “Draper” genome (Colle et al., 2019). The two *ACO* genes and ethylene signaling genes were identified from the “Powderblue” transcriptome generated in house (Wang and Nambeesan, *in preparation*; Supplementary Table 1). Three reference genes, *UBIQUITIN-CONJUGATING ENZYME* (*UBC28*), *RNA HELICASE-LIKE* (*RH8*), and *CLATHRIN ADAPTER COMPLEXES MEDIUM SUBUNIT FAMILY PROTEIN* (*CACSa*; Vashisth et al., 2011), were used to normalize the expression of the target gene. Changes in transcript abundance of ethylene biosynthesis and signaling genes were quantified as described previously (Rieu and Powers, 2009).

### Statistical Analyses

Statistical analyses were performed using JMP Pro 14 (SAS Institute Inc., Cary, NC, 1989–2021) and R-studio 2021 (R Core 2021, Vienna, Austria). If ANOVA was significant, it was followed by mean separation using Tukey’s Honest Significant Difference (HSD) test (*α* = 0.05) to determine changes across developmental stages and among cultivars.

## Results

### Respiratory Climacteric During Blueberry Ripening

**Figure 1:**
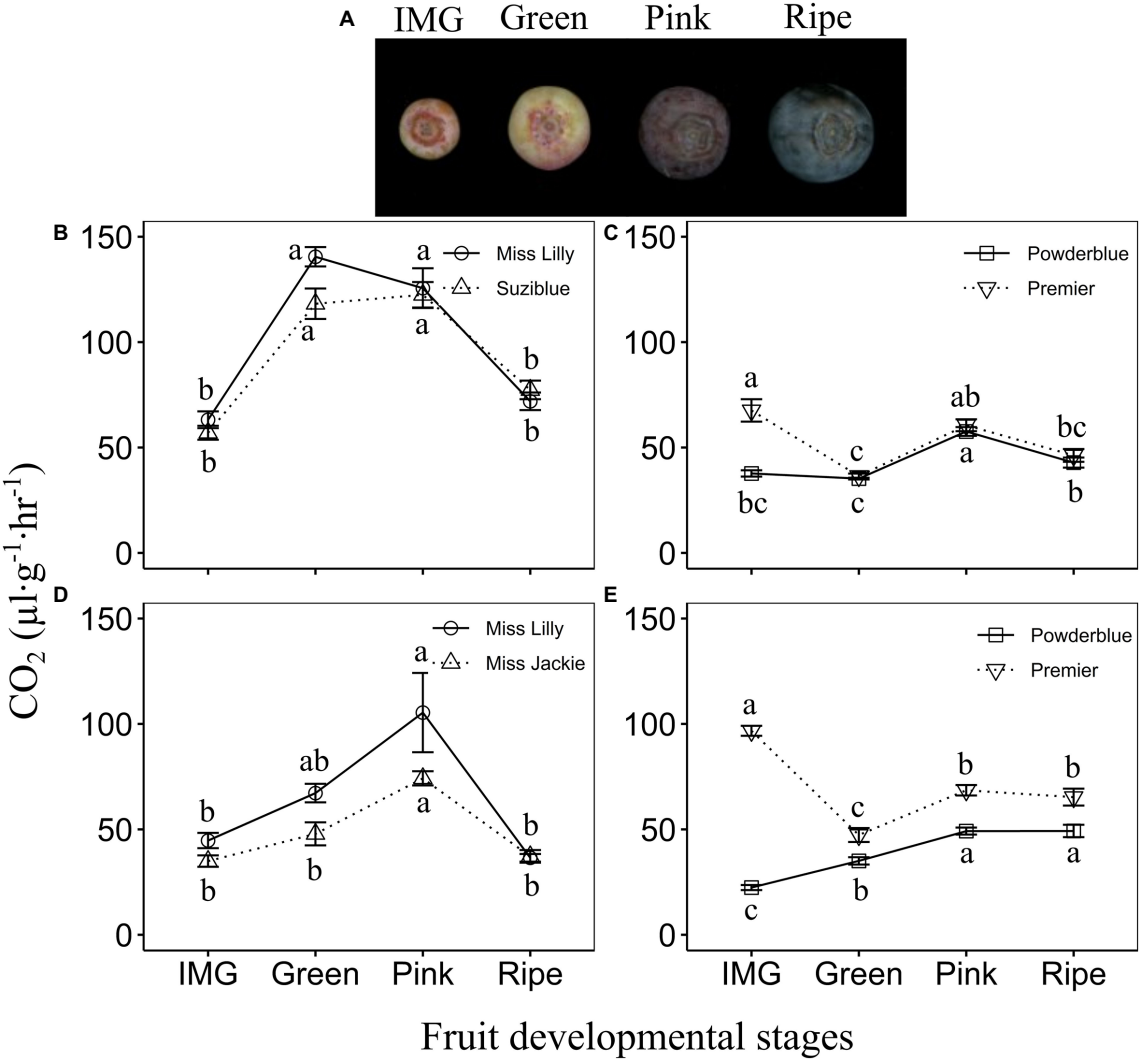
Fruit developmental stages **(A)**. Respiratory rate in southern highbush **(B,D)** and rabbiteye **(C,E)** blueberry cultivars during fruit ripening in 2017 **(B,C)** and 2018 **(D,E)**. IMG: Immature Green. Different letters indicate that the means are significantly different at different fruit stages within a given cultivar based on ANOVA and Tukey’s HSD (*α* = 0.05).

Increase in the rate of respiration was detected during fruit ripening in southern highbush and rabbiteye blueberry genotypes (Figure 1; Supplementary Figure 1). The southern highbush cultivars, Ms. Lilly and Suziblue (2017), displayed a sharp increase in respiration by 2-fold between the IMG and Green stages and these levels remained high during the Pink stage before declining by the Ripe stage (Figures 1A,B). Beyond the Ripe stage, levels remained low or decreased further during 8 and 21 days of postharvest storage (Supplementary Figure 1A). Similarly, in 2018, the rate of respiration increased during later stages of fruit development reaching the maximum value at the Pink stage (2-fold higher than that at the IMG stage), and declining thereafter (Figure 1D; Supplementary Figure 1C). In addition, several other southern highbush blueberry cultivars displayed similar patterns of increase in the rate of respiration across 2 years of evaluation (Supplementary Figures 1A,C). Generally, peak rate of respiration was noted at the Pink stage across these cultivars. Further, they displayed a decline in the rate of respiration from Pink to the Ripe stage by 1.4–2.9-fold, and these levels remained either similar or declined further during postharvest storage depending on the cultivar (Supplementary Figures 1A,C).

In the rabbiteye blueberry cultivar., Powderblue, the rate of respiration was highest during the S2 and S3 stages of early fruit development and decreased by 2-fold during the IMG stage (Supplementary Figure 1B). In another rabbiteye blueberry cultivar., Premier, the rate of respiration was higher during S3 and IMG stages but declined by 1.7-fold at the Green stage (Figure 1C; Supplementary Figure 1B). In “Powderblue,” respiration increased by over 1.6-fold from Green to the Pink stage after which it declined at the Ripe stage to levels noted in the IMG fruit (Figure 1C). Further, these levels were maintained during postharvest storage (Supplementary Figure 1B). In “Premier,” rate of respiration increased at the Pink stage by 1.7-fold from the Green stage (Figure 1C). Across four other cultivars tested in 2017, the respiration rate increased by up to 2.6-fold between the IMG and Pink stages (Supplementary Figure 1B). Across these cultivars, the highest rate of respiration during late fruit development was noted at the Pink stage. Similar patterns in the rate of respiration were observed in 2018 in “Powderblue” and “Premier,” except that the rate did not decline significantly after the Pink stage (Figure 1E; Supplementary Figure 1D). Four other cultivars evaluated in 2018 displayed respiration patterns similar to that observed in 2017 (Supplementary Figures 1B,D).

At the Pink stage, when respiration rate was the highest, no cultivar differences were observed in southern highbush blueberry, in both years (Figures 1B,D; Supplementary Figures 1A,C). In rabbiteye cultivars in 2017, at the Pink stage, the cultivars Alapaha, Brightwell, and Krewer, displayed higher rates of respiration than in “Powderblue” and “Premier” (Figure 1C; Supplementary Figure 1B). In 2018, “Brightwell” displayed a higher rate of respiration than that in “Powderblue” at the Pink stage (Supplementary Figure 1D). Generally, southern highbush blueberry genotypes displayed higher rates of respiration than the rabbiteye blueberry genotypes (Supplementary Figure 3A). In 2017, the highest rate of respiration noted at the Pink stage ranged between 122 and 151 μl·g^−1^·h^−1^ in southern highbush blueberry and between 58 and 80 μl·g^−1^·h^−1^ in rabbiteye blueberry (Supplementary Figures 1A,B). In 2018, differences between the two types of blueberry were less evident with the southern highbush cultivars displaying 74–105 μl·g^−1^·h^−1^ and rabbiteye cultivars between 50 and 74 μl·g^−1^·h^−1^ of CO_2_ evolution (Supplementary Figures 1C,D, 3B).

### Rate of Ethylene Evolution During Blueberry Fruit Ripening

**Figure 2:**
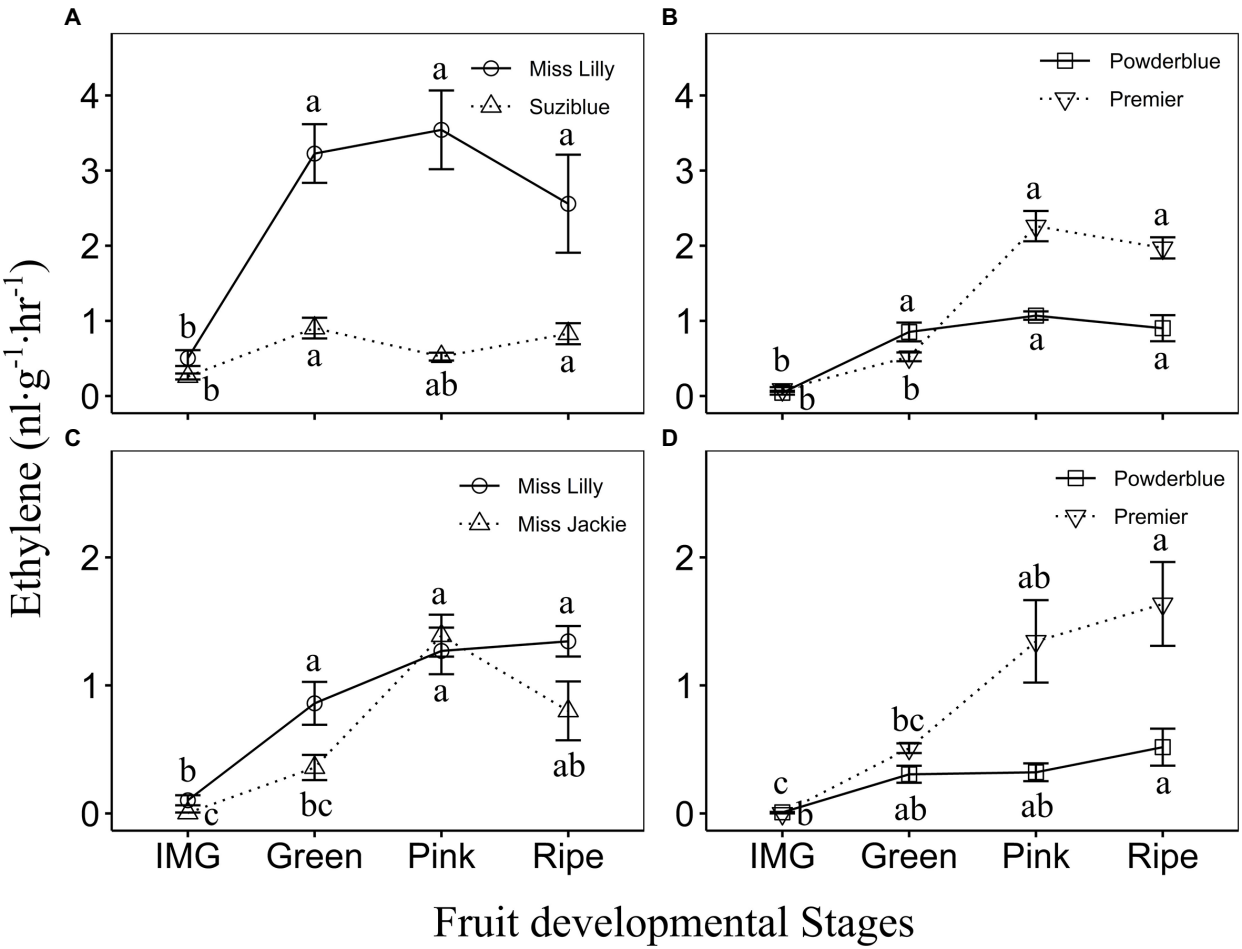
Ethylene evolution in southern highbush **(A,C)** and rabbiteye **(B,D)** blueberry cultivars during fruit ripening in 2017 **(A,B)** and 2018 **(C,D)**. IMG, Immature Green. The different letters indicate the means are significantly different at different fruit stages within a given cultivar according to ANOVA and Tukey’s HSD (*α* = 0.05). Note that the *Y*-axis scales are different for 2017 **(A,B)** and 2018 **(C,D)** data.

Over 2 years of study and across different cultivars of rabbiteye and southern highbush blueberry, the rate of ethylene evolution ranged from 0 to 5.3 nl·g^−1^·h^−1^ during fruit development (Figure 2; Supplementary Figure 2). In 2017, two southern highbush blueberry cultivars tested, displayed increase in ethylene evolution between IMG and Green stages (Figure 2A). The magnitude of increase in ethylene evolution was greater in “Miss Lilly” compared to “Suziblue” with 6.4-fold and a 3.5-fold increase, respectively between the IMG and Green stages (Figure 2A). It remained high during the rest of fruit development until the Ripe stage and then declined during postharvest storage to levels observed at the IMG stage (Figure 2A; Supplementary Figure 2A). In 2018, the rate of ethylene evolution in “Miss Lilly” was 8.4-fold higher between the IMG and Green Stage and remained high until the Ripe stage (Figure 2C). In “Miss Jackie” it was 4-fold higher between the Green and Pink stages and remained high until the Ripe stage (Figure 2C). In these two cultivars ethylene levels declined during postharvest storage, but in “Miss Jackie” this decrease was evident only at around 3 weeks of postharvest storage (Supplementary Figure 2C). In 2018, ethylene levels were measured in two additional cultivars. The rate of ethylene evolution increased by up to 2.4-fold and 2.5-fold between IMG and Green stages in “Emerald” and “Rebel,” respectively (Supplementary Figure 2C). In “Rebel,” it remained high during the rest of the fruit development until the Ripe stage, whereas in “Emerald” levels continued to increase from the Green stage until the Ripe stage (Supplementary Figure 2C). In both cultivars, ethylene declined during postharvest storage (Supplementary Figure 2C).

Among rabbiteye blueberry genotypes in 2017, ethylene evolution increased in “Powderblue” fruit between the IMG and Green stage by 19.5-fold and remained high for the rest of fruit development including during postharvest storage (Figure 2B and Supplementary Figure 2B). In “Premier,” ethylene evolution rate increased between the Green and Pink stages by 4.3-fold and remained high throughout ripening and early postharvest storage (Figure 2B; Supplementary Figure 2B). In “Titan” and “Brightwell,” the rate of ethylene evolution increased by 3- and 4.2-fold between Pink and Ripe stages and generally continued to remain high during most of postharvest storage (Supplementary Figure 2B). In 2018, the rate of ethylene evolution in rabbiteye cultivars, Powderblue, Premier, and Brightwell increased gradually over fruit development with the highest rate recorded at the Ripe stage and remained largely constant during postharvest storage (Figure 2D; Supplementary Figure 2D). In “Titan,” ethylene evolution displayed an increasing trend during ripening with the highest rate detected during postharvest storage (Supplementary Figure 2D).

Overall, differences in ethylene evolution were noted across southern highbush blueberry cultivars in both years (Supplementary Figures 2A,C). For example, in 2017, “Miss Lilly” had 4.3-fold higher rate of ethylene evolution compared to “Suziblue” at the peak production stage and in 2018, “Emerald” had up to 2.8-fold lower ethylene evolution compared to the three other cultivars evaluated at the Pink stage (Supplementary Figure 2A). Among rabbiteye cultivars in 2017, the rate of ethylene evolution at the Ripe stage in “Titan” was higher by 2.7–5.9-fold than in the other three cultivars (Supplementary Figure 2B). Also, “Premier” displayed higher ethylene evolution than “Powderblue” at the Pink stage. In 2018 too, “Premier” displayed higher ethylene production than “Powderblue” at the Pink (4.2-fold) and Ripe (3.2) stages, and with “Brightwell” at the Pink (4.7-fold) stage (Supplementary Figure 2C). Overall, differences in ethylene evolution between southern highbush and rabbiteye blueberry types were not apparent (Supplementary Figures 3C,D).

### Carbon Dioxide and Ethylene Evolution in Response to Ethylene Treatment

Among the rabbiteye blueberry cultivars tested, Premier and Powderblue displayed consistent differences in ethylene evolution during fruit ripening and were chosen for further analysis. Effects of ethylene treatment on the rate of respiration and ethylene release were evaluated in detached fruit. There were no significant differences in the amount of CO_2_ released after either Green or Pink stage fruit of “Premier” were treated with 1 and 10 ppm ethylene, in comparison to the control (Supplementary Figures 4A,B). Similarly, ethylene treatment did not alter the rate of respiration in “Powderblue” fruit treated at the Pink stage (Supplementary Figure 4C). Additionally, there were no differences in ethylene evolution following treatment of Green or Pink stage fruit of “Premier” with 1 and 10 ppm ethylene (Supplementary Figures 4D,E). Pink fruit of “Powderblue” similarly treated with 1 and 10 ppm ethylene did not display significant differences in ethylene evolution in comparison to the control (Supplementary Figure 4F).

### Changes in ACC Concentration During Rabbiteye Blueberry Fruit Development and in Response to Ethephon

**Figure 3:**
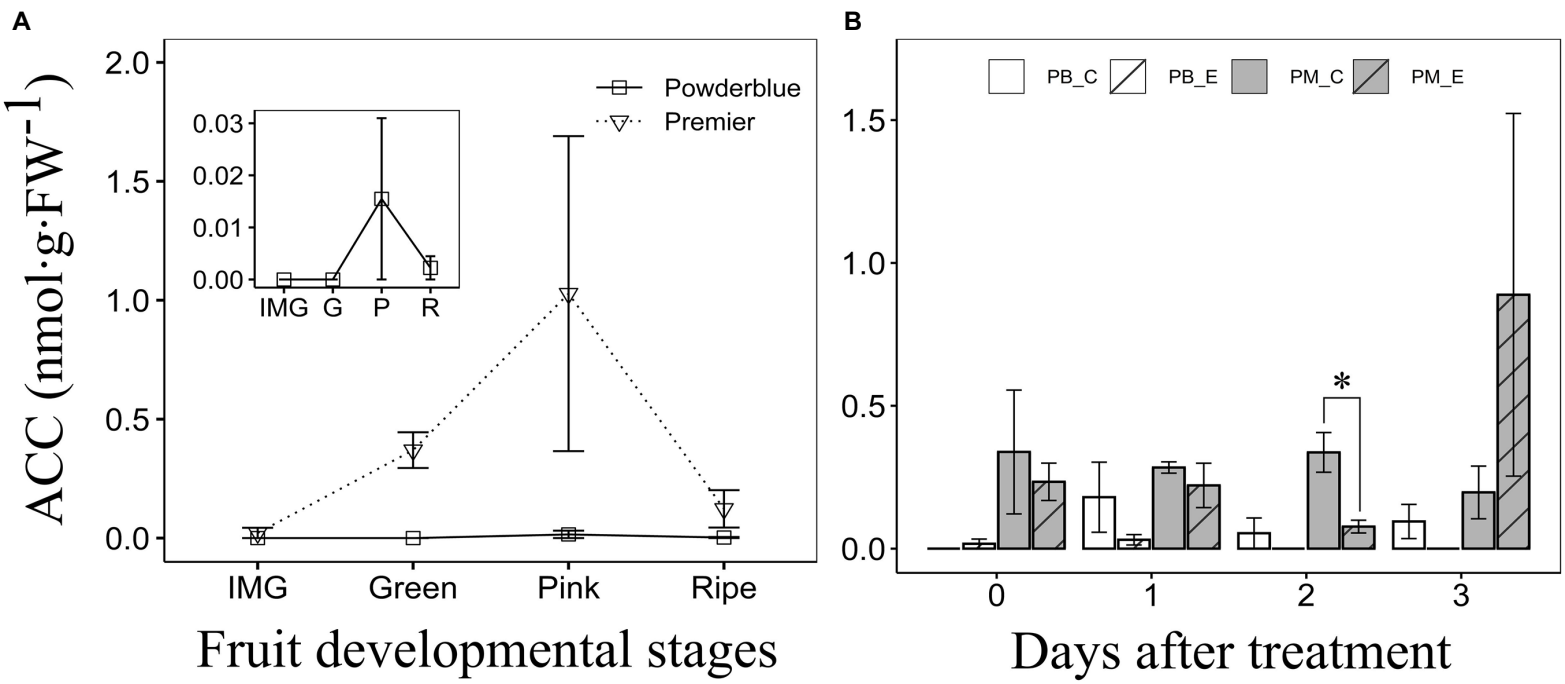
Concentration of 1-aminocyclopropane-1-carboxylic acid (ACC) during fruit ripening **(A)** and after treatment with water (Control) and 250 ppm ethephon in “Premier” and “Powderblue” **(B)**. Inset shows ACC concentration in “Powderblue” **(A)**. IMG, immature green; G, Green; P, Pink; R, Ripe; PB_C, Powderblue control; PB_E, Powderblue ethephon; PM_C, Premier control; and PM_E, Premier ethephon. No significant differences in ACC concentration across fruit developmental stages in a given cultivar were detected using ANOVA. Data represent mean ± SE. Asterisk indicates that the means are significantly different between treatments (control and ethephon), within a genotype at a given stage, according to *t*-test (*α* = 0.05).

Concentration of ACC was determined during fruit development in “Premier” and “Powderblue” (Figure 3A). ACC concentration was highly variable and generally below 1 nmol·g^−1^ (FW; Figure 3A). During fruit ripening, ACC concentration tended to increase between IMG and the Pink stages and then declined at the Ripe stage. However, ANOVA did not indicate significant differences across developmental stages in either cultivar (Figure 3A). Overall, ACC concentration was 1.6-fold higher in “Premier” than in “Powderblue” (Figure 3A). Treatment with the ethylene-releasing compound, ethephon, transiently reduced ACC concentration at 2 days after treatment in “Premier” by 4.4-fold. It did not influence ACC concentration in “Powderblue” (Figure 3B).

### Transcript Abundance of *ACS* and *ACO* During Fruit Development

**Figure 4:**
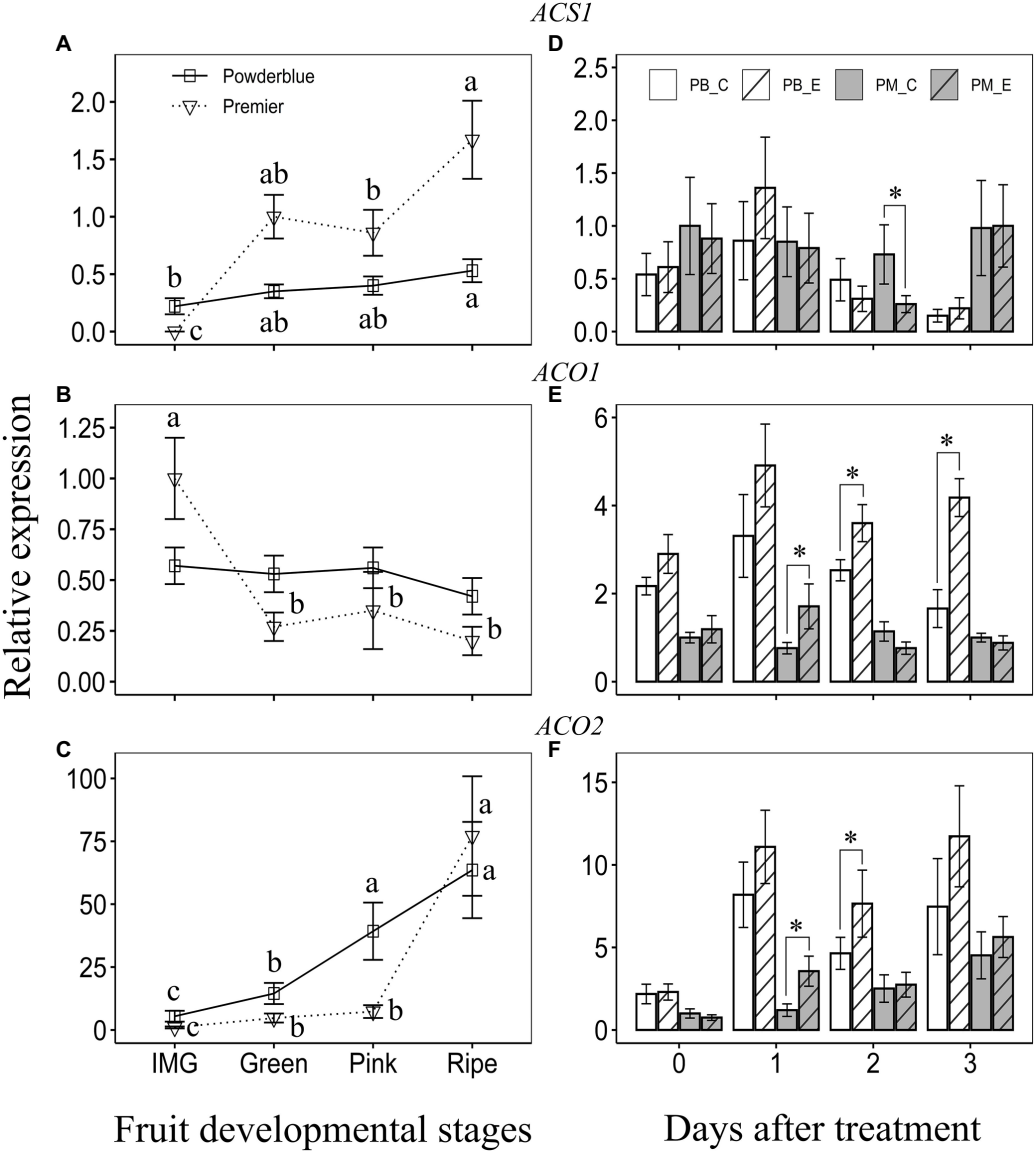
Transcript abundance of ethylene biosynthesis genes: 1-aminocyclopropane-1-carboxylic acid synthase (*ACS*; **A,D**), and 1-aminocyclopropane-1-carboxylic acid oxidase (*ACO*), *ACO1* **(B,E)**, and *ACO2* **(C,F)** during ripening (left panels) and treatment with water (control) and 250 ppm ethephon (right panels). IMG, Immature Green; PB_C, Powderblue control; PB_E, Powderblue ethephon; PM_C, Premier control; and PM_E, Premier ethephon. Different letters above symbols indicate that the means are significantly different across fruit developmental stages (within a cultivar) according to ANOVA and Tukey’s HSD (*α* = 0.05). Asterisk indicates the means are significantly different between treatments (control and ethephon), within a cultivar and given date, according to *t-*test (*α* = 0.05).

Transcript abundance of ethylene biosynthesis genes during fruit development was investigated in “Premier” and “Powderblue” (Figure 4). Among the five *ACS* genes analyzed, *ACS1* displayed highest abundance (based on Ct values). Transcript abundance of *ACS1* increased gradually over fruit development in “Powderblue” being 2.4-fold greater at the Ripe stage than during the IMG stage (Figure 4A). In “Premier,” it increased greatly between the IMG (undetectable) and Green stages and again between Pink and Ripe stages by 1.9-fold (Figure 4A). Generally, transcript abundance of *ACS1* in “Premier” was 2- to 3-fold higher than in “Powderblue” during later stages of fruit development (Figure 4A). Transcript abundance of four other *ACS* genes, *ACS2-5*, did not change significantly during fruit development in either cultivar and was not different between the two cultivars (Supplementary Figure 5).

Transcript abundance of *ACO1* was not altered during fruit development in “Powderblue” but declined by approximately 3.7-fold between IMG and Green stages in “Premier” (Figure 4B). Transcript abundance of *ACO2* gradually increased during fruit development in “Powderblue” by 11.6-fold between the IMG and Ripe stage (Figure 4C). In “Premier,” it increased 4.7-fold between IMG and Green and again by 10.6-fold between Pink and Ripe stages (Figure 4C). At the Green and Pink stages, transcript abundance of *ACO2* was generally higher in “Powderblue” than in “Premier” by up to 3-fold (Figure 4C).

### Effect of Ethephon on Transcript Abundance of Ethylene Biosynthesis-Related Genes

Ethephon treatment transiently reduced *ACS1* transcript abundance at 2 days after treatment in “Premier” by 2.8-fold (Figure 4D). It did not alter *ACS1* transcript abundance in “Powderblue” (Figure 4D). Further, transcript abundance of *ACS2* and *ACS3* were unaffected by ethephon treatment (Supplementary Figures 5E,F). *ACS4* transcript abundance significantly increased at 1 day after treatment in “Premier” and that of *ACS5* was reduced at 3 days after treatment in “Powderblue” (Supplementary Figures 5G,H). Transcript abundance of *ACO1* and *ACO2* increased in response to ethephon treatment at 1 day after treatment by up to 2.2- and 3-fold, respectively, in “Premier” (Figures 4E,F). Ethephon treatment in “Powderblue” also resulted in increased transcript abundance of *ACO1* at 2 (1.4-fold) and 3 days (2.5-fold) after treatment, and of *ACO2* at 2 days (1.6-fold) after treatment (Figures 4E,F).

### Transcript Abundance of Ethylene Signaling Genes During Fruit Development

**Figure 5:**
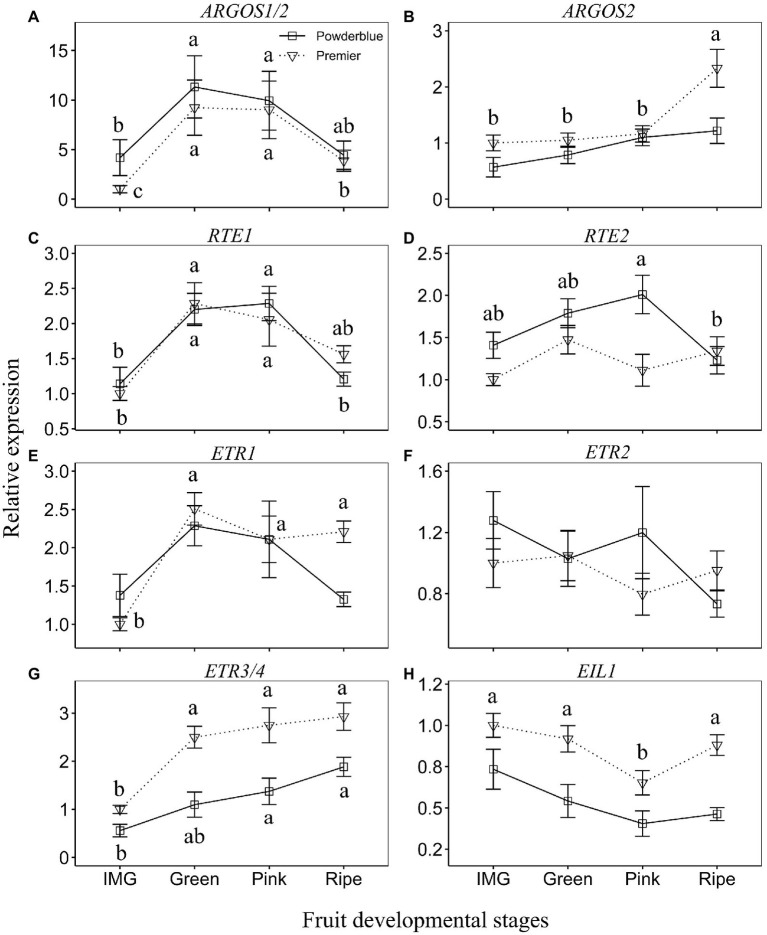
Transcript abundance of ethylene signaling genes during fruit development. **(A)** *ARGOS1/2*, **(B)** *ARGOS2*, **(C)** *RTE1*, **(D)** *RTE2*, **(E)** *ETR1*, **(F)** *ETR2*, **(G)** *ETR3/4*, and **(H)** *EIL1*. *ARGOS: AUXIN REGULATED GENE INVOLVED IN ORGAN SIZE; RTE: REVERSION TO ETHYLENE SENSITIVITY; ETR: ETHYLENE RECEPTOR; EIL1: ETHYLENE INSENSITIVE 3 – LIKE1*. The different letters indicate the means are significantly different across fruit developmental stages within a given cultivar according to ANOVA and Tukey’s HSD (*α* = 0.05).

The transcript abundance of *AUXIN REGULATED GENE INVOLVED IN ORGAN SIZE* (*ARGOS1/2*) increased by 2.7-fold in “Powderblue” and 9.2-fold in “Premier” between IMG and Green stages, remained high during the Pink stage and then declined at the Ripe stage (significantly in “Premier”; Figure 5A). The expression of *ARGOS2* increased by 2-fold between Pink and Ripe stages in “Premier” (Figure 5B). Transcript abundance of *REVERSION TO ETHYLENE SENSITIVITY1* (*RTE1*) was higher than that of *RTE2* (Figures 5C,D). The expression patterns of the *RTE* genes were similar in both cultivars, they increased by about 2-fold between IMG and Green stages, and declined at the Ripe stage (Figure 5C). The expression of *RTE2* declined by 1.6-fold between the Pink and Ripe stage in “Powderblue” but was not altered in “Premier” (Figure 5D). Transcript abundance of *ETHYLENE RECEPTOR1* (*ETR1*) increased 2.5-fold between IMG and Green stages and remained constant throughout ripening in “Premier” but it was not altered in “Powderblue” (Figure 5E). Overall transcript abundance of *ETR2* did not exhibit any changes during ripening in both the cultivars (Figure 5F). The transcript abundance of *ETR3/4* in both cultivars increased during ripening with a 2.5-fold increase in “Premier” between IMG and Green stages and in “Powderblue” between IMG and Pink stages (Figure 5G). The expression of *ETHYLENE INSENSITIVE 3–LIKE1* (*EIL1*) transiently decreased at the Pink stage in “Premier” and was not altered in “Powderblue” (Figure 5H).

### Effect of Ethephon on Transcript Abundance of Ethylene Signaling-Related Genes

**Figure 6:**
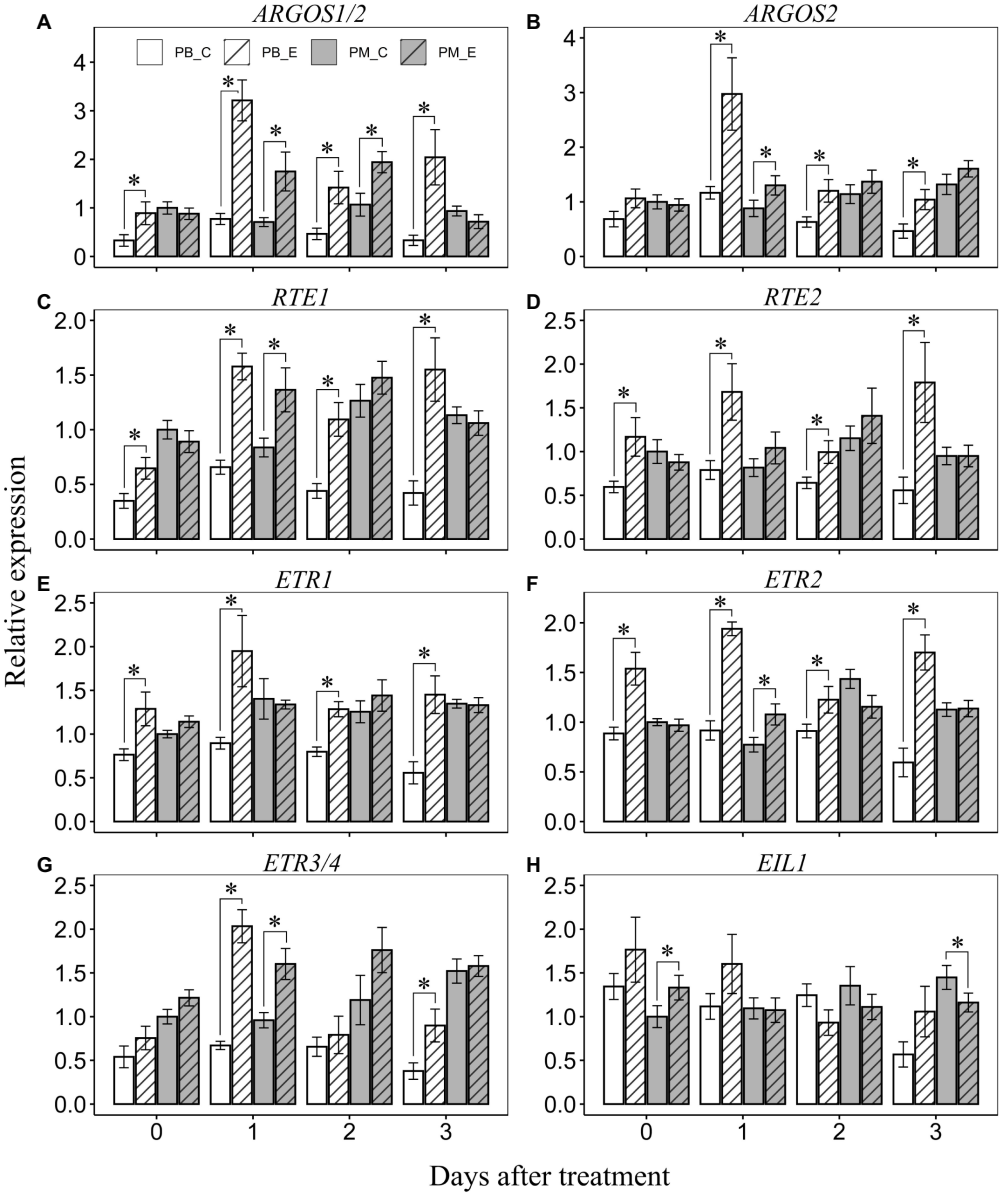
Transcript abundance of ethylene signaling genes after application of ethephon and control at 0, 1, 2, and 3 days after treatment. **(A)** *ARGOS1/2*, **(B)** *ARGOS2*, **(C)** *RTE1*, **(D)** *RTE2*, **(E)** *ETR1*, **(F)** *ETR2*, **(G)** *ETR3/4*, and **(H)** *EIL1*. *ARGOS*, *AUXIN REGULATED GENE INVOLVED IN ORGAN SIZE*; *RTE*, *REVERSION TO ETHYLENE SENSITIVITY*; *ETR*, *ETHYLENE RECEPTOR*; and *EIL1*, *ETHYLENE INSENSITIVE 3–LIKE1*. Asterisk indicates that the means are significantly different between treatments (control and ethephon) within a cultivar and given day according to *t*-test (*α* = 0.05).

Ethephon treatment enhanced the transcript abundance of *ARGOS1/2* in “Powderblue” compared to that in the control at all days after treatment. It was 2.7-, 4.2-, 3-, and 6.1-fold higher at 0, 1, 2, and 3 DAT compared to control (Figure 6A). In “Premier,” transcript abundance of *ARGOS1/2* was also enhanced by ethephon by 2.5- and 1.8-fold at 1 and 2 DAT compared to the control (Figure 6A). Transcript abundance of *ARGOS2* was enhanced by 2.6-, 1.9-, and 2.2-fold by ethephon in “Powderblue” at 1, 2, and 3 DAT, respectively (Figure 6B). It was 1.5-fold higher in response to ethephon in “Premier” at 1 DAT compared with control (Figure 6B). Transcript abundance of *RTE1* and *RTE2* was enhanced by ethephon treatment in “Powderblue” compared to the control at all days after treatment (Figures 6C,D). *RTE1* and *RTE2* transcript abundance was >3-fold higher by 3 DAT in response to the ethephon treatment in “Powderblue.” In “Premier,” only the transcript abundance of *RTE1* was 1.6-fold enhanced by ethephon at 1 DAT compared to the control (Figures 6C,D). After ethephon treatment, transcript abundance of the ethylene receptor genes, *ETR1*, *ETR2*, and *ETR3/4* was upregulated in “Powderblue” by up to 2.6-, 2.9-, and 3-fold, respectively between 1 and 3 DAT (Figures 6E–G). In “Premier,” *ETR2* and *ETR3* abundance was 1.4- and 1.7-fold upregulated, respectively, by ethephon treatment at 1 DAT (Figures 6E–G). The transcript abundance of *EIL1* was higher in “Premier” by 1.3-fold at 0 DAT and reduced by 1.2-fold at 3 DAT (Figure 6H).

## Discussion

Climacteric fruit ripening is characteristically associated with an increase in the rate of respiration at the onset of the ripening phase of fruit development (Giovannoni, 2004; Cherian et al., 2014). This increase in the respiration rate is concomitant with extensive metabolic changes which include synthesis and accumulation of pigments, fruit softening, and accumulation of a range of secondary metabolites including volatiles, which together constitute the respiratory syndrome. The primary cause for the respiratory climacteric is still not well understood, although several hypothesis such as a function in supporting energy requirements for carbon metabolism have been proposed (Beaudry et al., 1987). In contrast to climacteric fruits, the rate of respiration does not increase appreciably during the onset of ripening in non-climacteric fruits (Kays and Paull, 2004; Paul et al., 2012; McAtee et al., 2013). In the current study, rates of respiration increased during later stages of fruit development in southern highbush and rabbiteye blueberry cultivars, consistent with some previous reports (Ismail and Kender, 1969; Windus et al., 1976). Maximum rates of respiration were often observed during the Pink stage of fruit development (around 50–150 μl g^−1^ h^−1^) and were generally comparable or slightly higher than that reported in other climacteric fruits such as apple, tomato, and peach (around 50 μl g^−1^ h^−1^) (Andrews, 1995; Rudell et al., 2000). Importantly, the extent of increase by around 2-fold, compared to the pre-climacteric period, is within the respiratory climacteric increase reported in other climacteric fruits (Kays and Paull, 2004). Together, these data indicate that changes in respiration rate at the onset of ripening are consistent with climacteric ripening in blueberry.

Another characteristic feature associated with climacteric fruit ripening is an increase in ethylene evolution at the onset of ripening, concomitant with the respiratory climacteric (Paul et al., 2012; McAtee et al., 2013). In the current study, increase in ethylene evolution during late fruit development was consistently observed across multiple southern highbush and rabbiteye blueberry genotypes, albeit with varying levels. These data are consistent with those from a recent study which concluded that variation in ethylene evolution in highbush blueberry is primarily genotype-dependent (Farneti et al., 2022). Particularly, ethylene evolution increased multiple-fold during later stages in comparison to the pre-climacteric stages. The increase in ethylene evolution was concomitant with increase in the rate of respiration, generally reaching a maxima at the Pink or Ripe stages of fruit development. Ethylene evolution increased by up to 6-fold during this period. The peak values of ethylene evolution during ripening ranged between 0.5 to 5.3 nl·g^−1^·h^−1^ among cultivars, consistent with those reported for highbush blueberry (Farneti et al., 2022). The absolute values of ethylene release during climacteric fruit ripening vary considerably across fruit species, and across genotypes within a species (Biale et al., 1954; Burg and Burg, 1962; Kays and Paull, 2004; Minas et al., 2015; Pereira et al., 2020; Farneti et al., 2022). But, the values noted in the current study are comparable to those reported in other climacteric fruit species such as tomato, banana, apple, plum, and peach, and substantially greater than that noted in non-climacteric fruits such as grape, citrus, and strawberry (Biale et al., 1954; Bain, 1958; Iannetta et al., 2006; Sun et al., 2010). In fact, comparable levels (~2–8 nl·g^−1^·h^−1^) were reported during the onset (Turning stage) and later stages of ripening in the classical model for climacteric fruit, tomato (Nakatsuka et al., 1998; Yokotani et al., 2009). While these measurements indicate the rate of ethylene evolution from the organ, internal ethylene levels of around 0.1 ppm are suggested to be sufficient for eliciting an ethylene-dependent ripening response (Biale et al., 1954; Burg and Burg, 1962). Considering a conservative, estimated ratio of 2 ppm of internal ethylene per nL·g^−1^·h^−1^ of ethylene evolution (Burg and Burg, 1962), it is likely that fruit internal ethylene concentration in maturing blueberry fruit is sufficiently above the threshold for ethylene to regulate the progression of ripening. Hence, ethylene evolution during the onset of ripening in blueberry is consistent with a potential role for ethylene in regulating ripening. This conclusion is consistent with that from a recent study (Farneti et al., 2022).

ACS catalyzes the rate limiting step in ethylene biosynthesis, the conversion of SAM to ACC, and is encoded by a multi-gene family (Nakatsuka et al., 1998; Barry et al., 2000). In tomato, *SlACS1A* and *SlACS6* are expressed during early fruit development and their gene products aid in regulating autoinhibitory system 1 ethylene (Nakatsuka et al., 1998; Barry et al., 2000; Yokotani et al., 2009). During the transition from system 1 to autocatalytic system 2 ethylene, transcript abundance of *SlACS2* and *SlACS4* increases, potentially through ethylene-independent developmental, and through auto-catalytic, ethylene-dependent mechanisms (Yokotani et al., 2009). ACO catalyzes the terminal step in ethylene biosynthesis with the conversion of ACC to ethylene and does not appear to be rate limiting in fruits such as tomato, although transcript abundance of several *ACO* genes, *SlACO1* and *SlACO4*, is greatly enhanced at the onset of ripening and is subject to positive feedback regulation (Nakatsuka et al., 1998).

In “Premier,” a large increase in *ACS1* transcript abundance was noted between IMG and Green stages, preceding the increase in ethylene evolution, suggesting association of *ACS1* with increase in ethylene evolution during late fruit development. In “Powderblue,” the cultivar with lower ethylene evolution, *ACS1* transcript abundance did not change substantially during most of fruit development but was still higher at the Ripe stage compared to that at the IMG stage. Although not significant, ACC abundance increased transiently at the onset of ripening and was substantially higher in “Premier” than in “Powderblue” during this period. Higher ACC concentration and subsequently greater ethylene evolution in “Premier” were therefore associated with a larger increase in transcript abundance of *ACS1*. Transcript abundance of *ACO2* increased greatly in both cultivars during later stages of ripening. Together, these data suggest that *ACS1* and *ACO2* mediate the developmental increase in ethylene evolution during fruit ripening in blueberry with *ACS1* being rate limiting in blueberry.

Climacteric ripening responses regulated by ethylene are often associated with a transition from system 1 to autocatalytic system 2 ethylene. In fruits such as strawberry, loquat, and a non-climacteric plum genotype, lack of an autocatalytic ethylene response due to negative feedback was associated with non-climacteric behavior (Atta-Aly et al., 2000). In the current study, application of ethephon resulted in a transient decrease in *ACS1* transcript abundance accompanied by a decrease in ACC concentration, suggesting the lack of an autocatalytic system 2 ethylene response at the level of ACC synthesis in blueberry. However, transcript abundance of *ACO1* and *ACO2* were enhanced in response to ethephon within 1 day in “Premier” and at 2–3 days after treatment in “Powderblue.” These data indicate that *ACO* is inducible by ethylene and that a potential autocatalytic response is functional at the level of ACO.

Ethylene perception occurs through transmembrane receptors (ETR1; ETR2; EIN4; ERS1; and ERS2 in *Arabidopsis thaliana*) which are mainly localized to the endoplasmic reticulum and function as negative regulators of ethylene signaling. In the absence of ethylene, ethylene receptors activate CONSTITUTIVE TRIPLE RESPONSE 1 (CTR1), a kinase and negative regulator of ethylene signaling. In the absence of ethylene, CTR1 phosphorylates and inactivates EIN2, but in its presence, CTR1 is inactivated resulting in the cleavage of the C-terminal of EIN2 and its translocation to the nucleus where it stabilizes transcription factors such as EIN3 and EIN3-like (EIL1-5), which in-turn promote expression of ethylene dependent genes. Alternatively, EIN2 may bind to the mRNA of EIN3 binding F-box proteins, *EBF1* and *EBF2*, and down-regulate their translation (Li et al., 2015; Merchante et al., 2015).

Transcript abundance of ethylene receptors is often enhanced during the initiation and progression of ripening, particularly in climacteric fruits, and in response to ethylene (Nakatsuka et al., 1998; El-Sharkawy et al., 2007; Kevany et al., 2007; Farcuh et al., 2019). Ethylene receptor transcript abundance changes in non-climacteric fruits appear to be more variable. In non-climacteric fruits such as loquat, transcript abundance of ethylene receptor genes in the peel declined during the period when ethylene evolution was high. In the pulp, it was lower during early ripening and increased at later stages (Alos et al., 2017). In strawberry, transcript abundance of several ethylene receptor genes was upregulated during ripening and displayed ethylene inducibility (Trainotti et al., 2005). In plum, ethylene receptor transcript abundance increased substantially during fruit development in a climacteric genotype but was lower in a semi-climacteric genotype (El-Sharkawy et al., 2007). In the current study, transcript abundance of multiple genes (*ETR2* and *ETR3/4*) potentially coding for ethylene receptors was elevated in blueberry fruit during later stages of fruit development concomitant with increase in ethylene evolution. *ETR2* and *ETR3/4* displayed over 2-fold increase in transcript abundance (in ‘Premier’) during this period. Further, application of ethephon increased the transcript abundance of *ETR1*, *ETR2* and *ETR3/4* in ‘Powderblue’ and that of *ETR2* and *ETR3/4* in ‘Premier’. The ethylene perception and signaling model indicates that ethylene receptors function in negative regulation of its signaling. Hence, increase in their transcript abundance during fruit ripening and their ethylene-inducibility in blueberry suggest negative feedback, de-sensitization and fine regulation of ethylene responses. However, ethylene receptors are also regulated by extensive post-translational modifications such as degradation upon ligand (ethylene) binding and changes in phosphorylation status (Chen et al., 2007; Kevany et al., 2007; Kamiyoshihara et al., 2012). Hence, evaluation of their post-translational status is essential to determine the consequences of changes in ethylene receptor transcript abundance. Despite this, transcriptional inducibility of its receptors by ethylene is dependent on direct ethylene signaling, as it is altered in ethylene signaling mutants such as *ctr1-2* (O’Malley et al., 2005; Chang et al., 2013; Azhar et al., 2020). Hence, transcript abundance changes of ethylene receptors during ripening and their ethylene-inducibility in blueberry are consistent with functional ethylene signaling during fruit ripening.

*REVERSION TO ETHYLENE* and ARGOS are postulated to regulate sensitivity of ethylene signaling, specifically in de-sensitizing plant responses to ethylene (Resnick et al., 2006; Dong et al., 2008; Rai et al., 2015; Shi et al., 2015; Azhar et al., 2020). In Arabidopsis, transcript abundance of *RTE1* is upregulated in response to ethylene, a response mediated by EIN3, indicating a potential feedback-regulation mechanism to fine-tune ethylene responses (Chang et al., 2013; Rai et al., 2015; Azhar et al., 2020). In tomato, the *SlGR* (*GREEN RIPE*) gene, a member of the small RTE family, is ectopically expressed in the *green ripe* mutant resulting in reduced ethylene responsivity and a non-ripening fruit phenotype (Barry and Giovannoni, 2006). Although, *SlGR* transcript abundance is low in the fruit and not inducible by ethylene, that of *SlGRL1*, the homolog with highest identity with Arabidopsis *RTE1*, increases during fruit ripening and is inducible by ethylene (Ma et al., 2012). ARGOS is an ethylene-inducible gene that may interact with RTE1 and aid in de-sensitizing plants to ethylene (Rai et al., 2015). Ethylene induced increase in transcript abundance of *ARGOS* genes is decreased in mutants defective in ethylene signaling (e.g. *etr1-1* in Arabidopsis) indicating that a functional ethylene signaling pathway is essential in inducing this response (Rai et al., 2015), similar to that noted above for *RTE1* in Arabidopsis. In blueberry, transcript abundance of *RTE* and *ARGOS* genes increased during later stages of ripening, concomitant with changes in ethylene evolution. Transcript abundance of *ARGOS* and *RTE* genes was also upregulated in response to ethephon application in both cultivars, although it appeared to occur to a greater extent in “Powderblue.” Together, these responses further support the presence of a functional ethylene signaling program during the ripening stages of fruit development in blueberry. Hence, increase in ethylene evolution during later stages of fruit development concomitant with the respiratory climacteric is associated with functional ethylene signaling, supporting a role for ethylene in mediating climacteric ripening responses in blueberry.

This study clearly demonstrates changes in the rate of respiration and ethylene evolution consistent with climacteric fruit ripening across multiple blueberry genotypes. Increase in ethylene evolution during fruit ripening in blueberry is associated with altered transcript abundance of ethylene biosynthesis, perception and signaling-related genes indicating functional ethylene signaling during blueberry ripening. Hence, ethylene is likely to influence the fruit ripening syndrome in blueberry. However, the blueberry fruit does not display autocatalytic system 2 ethylene in relation to *ACS* transcript abundance and ACC accumulation during ripening, indicating partial uncoupling of climacteric responses from system 2 ethylene, and a potentially larger role for developmental regulation for ethylene synthesis during fruit ripening. Hence, we conclude that blueberry fruit display atypical climacteric responses with a role for ethylene-mediated regulation. Specific aspects of the ripening syndrome regulated by ethylene need to be carefully evaluated in the future to determine the contribution of ethylene to the maturation of the blueberry fruit. Further, considering that other phytohormones such as ABA may also regulate fruit ripening (Zifkin et al., 2012), interaction between these two phytohormones needs to be investigated.

## Data Availability Statement

The original contributions presented in the study are included in the article/Supplementary Material; further inquiries can be directed to the corresponding author.

## Author Contributions

Y-WW and SN conceived the study. Y-WW, AM, DSS, and SN designed the experiments. Y-WW, TA, AM, JD, and SN collected data and processed samples for ethylene and CO_2_ measurements, and gene expression analysis. H-JT and TA performed ACC quantification. Y-WW, TA, and JD performed ethephon applications. Y-WW, SN, and TA were involved in data analysis. Y-WW, TA, AM, DSS, and SN helped in preparation of figures and manuscript. All authors contributed to the article and approved the submitted version.

## Funding

This publication was partly supported by the U.S. Department of Agriculture’s (USDA) Agricultural Marketing Service through grant AM180100XXXXG014. Its contents are solely the responsibility of the authors and do not necessarily represent the official views of the USDA.

## Conflict of Interest

The authors declare that the research was conducted in the absence of any commercial or financial relationships that could be construed as a potential conflict of interest.

## Publisher’s Note

All claims expressed in this article are solely those of the authors and do not necessarily represent those of their affiliated organizations, or those of the publisher, the editors and the reviewers. Any product that may be evaluated in this article, or claim that may be made by its manufacturer, is not guaranteed or endorsed by the publisher.
