# Supplementary material for: Atypical Climacteric and Functional Ethylene Metabolism and Signaling During Fruit Ripening in Blueberry (Vaccinium sp.)
Source: Front Plant Sci. 2022 Jun 23;13:932642. doi: 10.3389/fpls.2022.932642 (PMC9260287; doi:10.3389/fpls.2022.932642)
Supplement: Supplementary file 1 [file Table_1.docx]

**Supplementary Table 1.** List of blueberry *ACS* and *ACO* genes and the primer sequences used in the quantitative PCR analyses.

Sequence source: Draper genome (Colle et al., 2019) (*ACS1*-*ACS5*) and Powderblue transcriptome (*ACO1*, *ACO2*, *ETR1*, *ETR2*, *ETR3&4*, *ARGOS1&2*, *ARGOS2*, *RTE1*, *RTE2*, *EIL1*)
