# Supplementary figures and images for: Atypical Climacteric and Functional Ethylene Metabolism and Signaling During Fruit Ripening in Blueberry (Vaccinium sp.)
Source: Front Plant Sci. 2022 Jun 23;13:932642. doi: 10.3389/fpls.2022.932642 (PMC9260287; doi:10.3389/fpls.2022.932642)

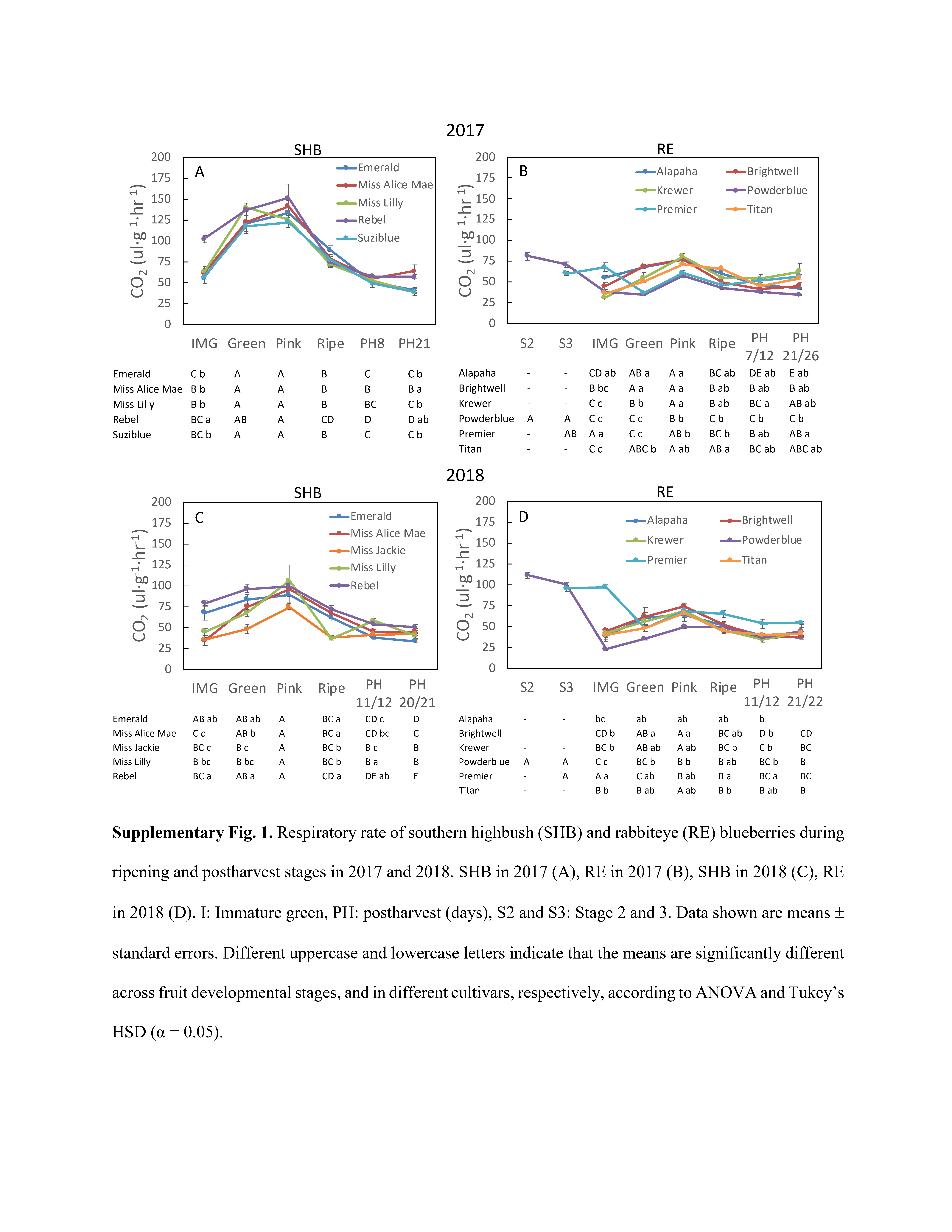

Supplement: Supplementary file 2 [file Image_1.TIFF]

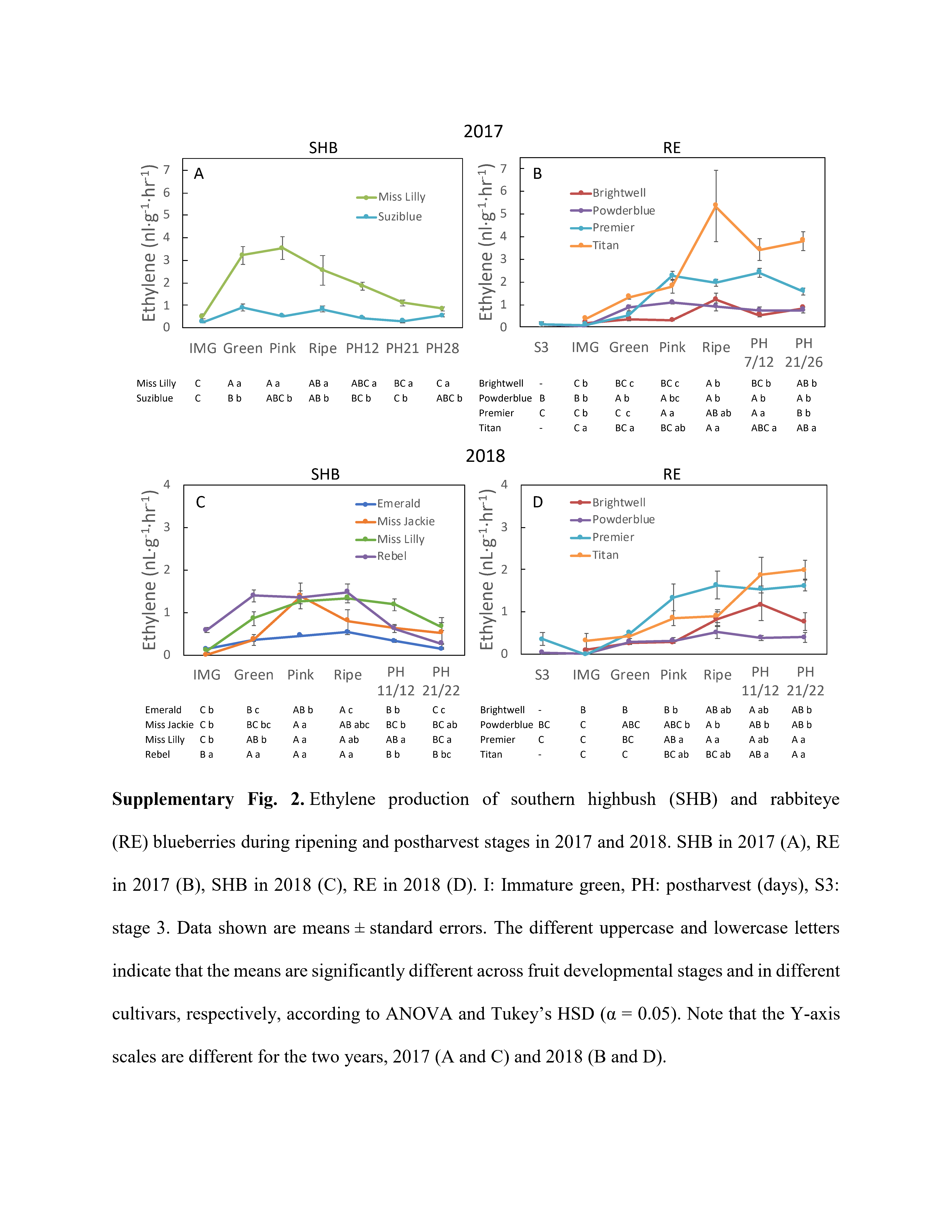

Supplement: Supplementary file 3 [file Image_2.TIFF]

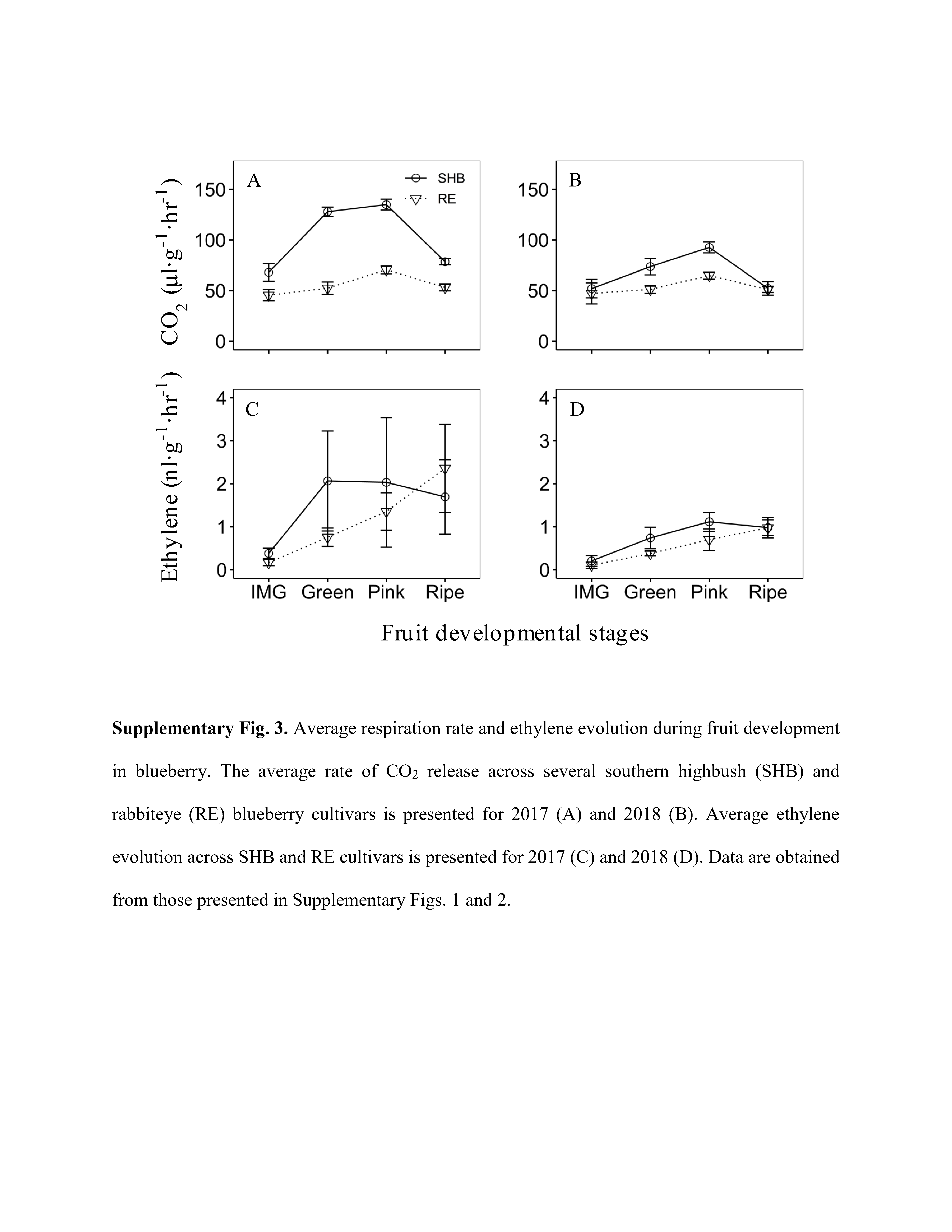

Supplement: Supplementary file 4 [file Image_3.TIFF]

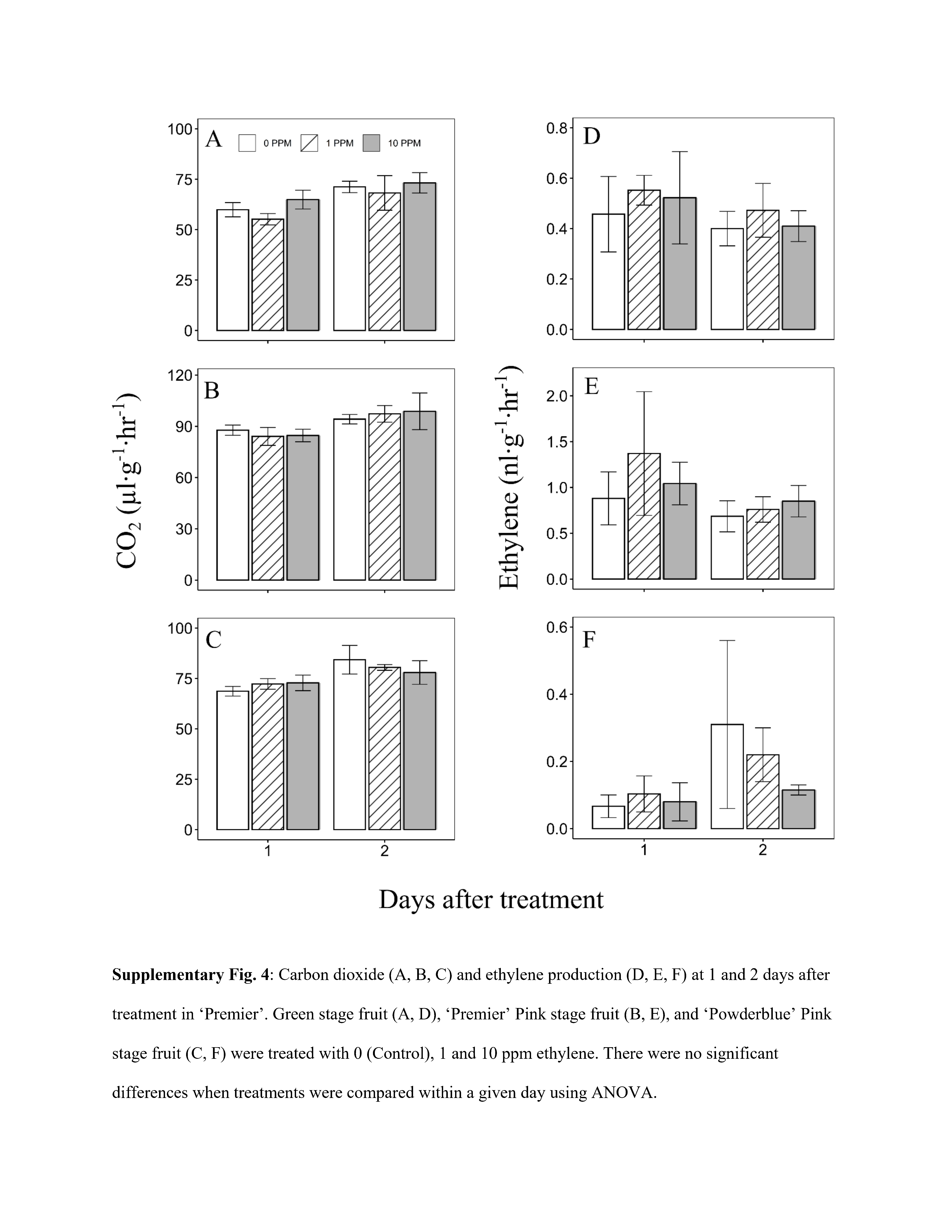

Supplement: Supplementary file 5 [file Image_4.TIFF]

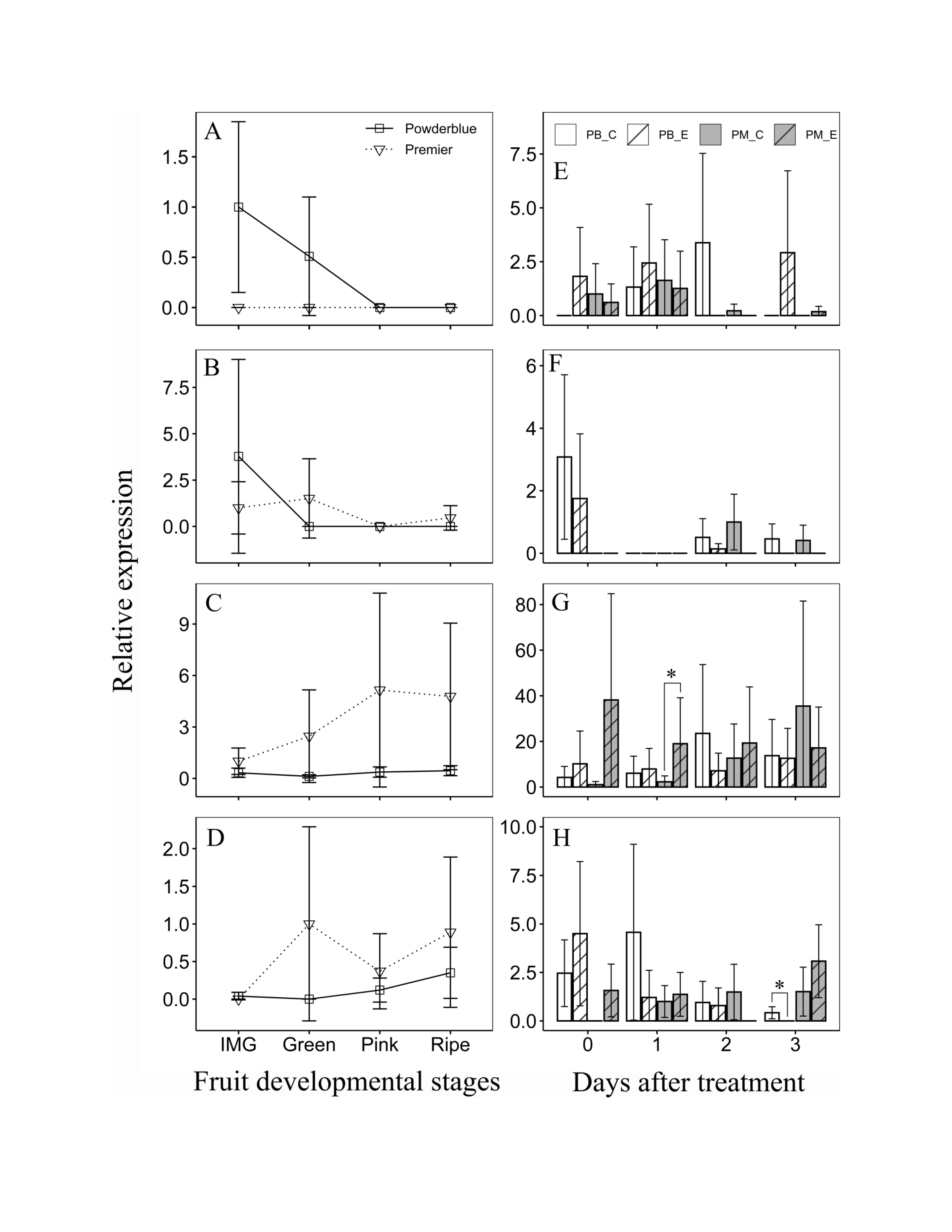

Supplement: Supplementary file 6 [file Image_5.TIFF]
